# Radiology education for medical students: a qualitative exploration of educational topics, teaching methods and future strategies

**DOI:** 10.1186/s12909-024-05879-0

**Published:** 2024-08-19

**Authors:** Frederike S. Harthoorn, Sascha W. J. Scharenborg, Monique Brink, Liesbeth Peters-Bax, Dylan Henssen

**Affiliations:** 1 Radboud University Nijmegen, Nijmegen, The Netherlands; 2 Department of Medical Imaging, Radboud University Medical Center, Geert Grooteplein Zuid 22, 6525 GA Nijmegen, The Netherlands

**Keywords:** Radiology education, Medical education, Medical school curriculum, Qualitative research

## Abstract

**Background:**

Imaging techniques play a central role in modern medicine and therefore it would be beneficial for all medical students to incorporate radiology education in medical school curricula. However, a formal undergraduate radiology curriculum with well-defined learning objectives remains lacking in The Netherlands. This study aims to qualitatively ascertain opinions from clinicians (radiologists and non-radiologists) with regard to radiology education in the medical school curricula, including topics, teaching methods and strategies.

**Methods:**

A qualitative study with in-depth semi-structured interviews was conducted. Inclusion was carried out until saturation was achieved, after which 2 additional interviews were held. Interviews were conducted using open-ended questions, following a predefined topic list. The constant comparative method was applied in order to include new questions when unexpected topics arose during the interviews. All interviews were transcribed verbatim and coded using a thematic analysis approach. Codes were organized into categories and themes by discussion between the researchers.

**Results:**

Forty-four clinicians were interviewed (8 radiologists, 36 non-radiologists). The three main themes that were derived from the interviews were: (1) expectations of indispensable knowledge and skills on radiology, (2) organization of radiology education within the medical curriculum and (3) promising educational innovations for the radiology curriculum. The qualitative study design provides more in-depth knowledge on clinicians’ views on educational topics.

**Conclusions:**

The themes and statements of this study provided new insights into educational methods, timing of radiology education and new topics to teach. More research is needed to gain consensus on these subjects and inclusion of the opinion of medical students with regard to radiology education is needed.

## Key points

• Radiology education in medical curricula was considered important by all interviewees

• Integrating radiology and anatomy in Longitudinal learning communities (LLC’s) could be a promising educational strategy

• Integration of ultrasound education in medical curricula should be investigated further

## Background

Imaging technologies play a central role in the practice of modern medicine. Therefore, it is not surprising that previous studies suggest that all medical students would benefit from (basic) knowledge concerning medical imaging technologies and radiology [24, 37, 63]. However, radiology education is not well integrated in the medical curricula [21, 25, 29, 37] and students’ lacking knowledge can be potentially dangerous [19, 63]. In turn, medical students (including interns) and residents reported a lack of confidence when interpreting radiology examinations, including (chest) radiographs [19, 48]. Moreover, lacking radiological knowledge was found to be correlated with an overutilization of medical imaging services [27], leading to increased societal healthcare-related expenses. Consequently, a need for radiology education in medical schools is recognized among teachers, medical students and curriculum designers [1, 29, 37, 42, 44, 47, 48, 52, 61]. Albeit, the learning objectives of such a radiology curriculum remains a topic of debate [61]. Therefore, proper identification of useful learning objectives for radiology education in medical curricula should be carried out [23, 54, 60, 61]. The first step of defining learning objectives is to determine which educational topics are important to teach [26, 34, 60, 5].

When defining these, it is important to identify the opinions of both clinicians (radiologists and non-radiologists) and medical students since both groups influence which topics are considered important to teach during medical school [39]. Opinions on this topic diverse, due to the fast technological developments in this broad field, which covers nearly every medical discipline for diagnostic and therapeutic purposes [23, 34, 63]. Several studies have previously aimed to determine radiology curriculum topics by questioning different groups of physicians (both radiologists and non-radiologists) and educational experts using questionnaires [32, 36, 48, 53, 53–55, 55, 61]. Overall, these studies provided lists of interpretative and non-interpretative skills that respondents agreed on what should be taught in medical school regarding radiology. The most commonly mentioned interpretative skill concerned the systematic approach of reading chest radiographs [32, 36, 48, 53, 53–55, 55]. Suggested non-interpretative skills were more diverse and included (a) the basic physical mechanisms of ionizing radiation, including knowledge on radiation risks [32, 36, 53, 53–55, 55, 61], (b) the principles of justification of procedures (e.g., knowing when to use intravenous contrast agents) [32, 36, 48, 53, 53–55, 55, 61], and comprehension of the role, indications and limitations of diagnostic imaging (and interventional) techniques [32, 36, 48, 53, 53–55, 55, 61].

Nevertheless, the aforementioned studies used a survey-based approach in which rather pre-determined information is collected from a large group of participants [32, 36, 48, 53, 53–55, 55]. This study aimed to build on this work by employing an inductive, qualitative approach, allowing for the opportunity to acquire participants’ opinions without any influence of preset questions and to explore these answers to gain more detailed information on a broad range of topics [22, 58]. Therefore, it is possible to gain a more accurate insight into the wide diversity of current ideas on education on imaging technology that are continuously changing. Limitations of qualitative research, on the other hand, concern the labor-intensive nature of such studies, which explains why in most fields, qualitative data are lacking. Also, qualitative data are more subjective than quantitative data as the interviewee has more control over the content of the data. Therefore, unnecessary quantification of qualitative data should be avoided as it falsely suggests objective, statistically proven results [10, 38].

Consequently, there is a recognized need for enhanced radiology education in medical schools among teachers, students, and curriculum designers. This study aimed to 1) Identify key topics that should be included in a radiology curriculum, 2) Determine effective teaching methods for radiology education and 3) Propose strategies for integrating radiology education into existing medical school curricula. Therefore, we qualitatively investigated the perspectives of clinicians (both radiologists and non-radiologists) on radiology education in medical curricula.

## Methods

### Design

An exploratory inductive qualitative study focusing on the role of radiology education in medical curricula was performed. A pragmatic qualitative approach was used with the aim to identify topics in radiology education that clinicians considered important to embed in the medical curriculum. A sample of clinicians involved in medical education in the Netherlands was asked to provide their insights using in-depth semi-structured interviews. Interviews were performed following an inductive iterative process using the constant comparative method [31]. This implies that if new topics arose during interviews, it was possible to explore these topics and thereby allowing new topics to be added to the interview guide during the experiment. The interview guide is provided in Table 1. After interviewing, a thematic approach was used to analyze the data.

**Table 1:** Interview guide used in this study

Part A	
Objective: To ascertain the desired foundational knowledge in the field of Radiology and Nuclear Medicine that a physician should possess upon completion of the Medicine training program	
***Sub questions***	- *What is considered fundamental knowledge regarding Radiology and Nuclear Medicine?* - *How can students improve their own knowledge regarding Radiology and Nuclear Medicine in light of their daily activities when working at your practice/department?* - *What knowledge do clinicians anticipate that students have upon completion of their medical education with regard to Radiology and Nuclear Medicine?*
**Part B**	
Objective: To ascertain the methods of teaching Radiology and Nuclear Medicine in medical curricula and to probe views and expectations of students with regard to this	
***Sub questions***	- *In what manner do you believe students acquire knowledge about Radiology and Nuclear Medicine within medical curricula?* - *What types of education should be provided?* - *What topics should be covered in this education?* - *What kind of educational sessions should be provided?* - *How do the clinicians expect the amount and level of detail of education on Radiology and Nuclear Medicine within the current program?* - *Is there any specific education on Radiology and Nuclear Medicine that clinicians feel is missing, inadequate or superfluous?*

Relevant scientific literature was reviewed on learning objectives and teaching methods in radiology education in medical school. After reviewing the available literature, two researchers (F.H. en D.H.) constructed a topic list. An inductive iterative interviewing process was carried out using the constant comparative method [51]. Therefore, new topics could be added to the topic list during the interviews.

### Participants

A list of eligible clinicians was constructed by reviewing hospitals and general practitioners within the training region of the university medical center in the east of The Netherlands (OpleidingsRegio Oost-Nederland). The contact person of each practice or department that provided a mandatory internship within the medical curriculum or an elective internship in radiology was contacted by e-mail in order to recruit eligible clinicians. Only clinical specialties embedding radiological imaging in their daily clinical practice were deemed eligible. Therefore, clinicians of the department of psychiatry, dermatology and ophthalmology were excluded from this study. The remaining clinicians were eligible if they participated in any medical curriculum in the Netherlands, regardless of being involved in the Bachelor’s or Master’s phase. Additionally, clinicians needed to be board-certified and actively working medical specialists, general practitioners or residents in radiology. Moreover, board-certified radiologists of the same training region who were involved in (any) medical curriculum in the Netherlands were included to provide more insight into what these “imaging experts” considered important to teach. Eligible clinicians were contacted by use of e-mail. After no initial response, the eligible clinicians were contacted again two weeks later. A third reminder was sent after a longer period of time, which varied from two weeks to three months. If no response was received, the participant was excluded from further inclusion.

### Ethical statement

This study was approved by the ethics committee of the Netherlands Association of Medical Education (NVMO, case number 2023.2.9). Before being interviewed, clinicians confirmed to participate in the study. Informed consent was obtained from all clinicians prior to the interview in which the clinicians consented to have the interview audio-recorded for further analyses. Moreover, all methods were carried out in accordance with relevant guidelines and regulations. All recorded data was stored on a secured disc, to which only one researcher (F.H.) had access. Transcribed data was stored and analyzed anonymously.

### Interviews

Individual semi-structured interviews were conducted by one of the researchers (F.H.). Clinicians decided in which way the interviews were held: in person, via electronic telecommunication software (i.e. Skype version 8.65.0.78; Skype Technologies, Luxembourg City, Luxembourg Palo Alto, CA, United States) or by telephone. In addition, four clinicians provided extensive answers to interview questions via e-mail. These data were also used in the data analysis. The interviews started with a short introduction of the research content followed by an open question on the participant’s thoughts on this matter. During the interviews, the interviewer used open-ended questions and encouraged the clinicians to speak openly and express their opinions, thoughts and considerations. The interviewer explained that there were no relations with the board of examiners, the university medical center educational board or the educational management team. In order to ensure reliable data, all interviews were audio-recorded and transcribed verbatim afterwards. Each transcript was thereafter analyzed using direct content analysis [30]. Starting after the first interview had taken place; transcriptions were coded line-by-line, through which a code list was created. Coding was continued after each interview. Inclusion of new participants was halted when no new topics and codes arose from this process, indicating that data saturation occurred. To confirm data saturation, two additional interviews were held. When confirmed, inclusion of new participants was stopped.

### Data analysis

The interview transcripts were analyzed qualitatively. The first four transcripts were independently analyzed by two researchers (F.H. and B.v.W.). Coding results were compared and discrepancies were resolved by discussion. If necessary, a third more experienced investigator (D.H.) could be asked to help resolve issues. Since there were no major discrepancies, further coding and analysis were carried out independently by one of the researchers (F.H.), who met periodically with one of the other researchers (D.H.) to discuss codes and themes until consensus was reached. The coding process was performed using Atlas.ti software, version 8.2.29.0 (ATLAS.ti Scientific Software Development GmbH, Berlin, Germany). The constructed codebook was organized into categories and themes which arose after discussion of all the different codes between two of the researchers (F.H. and D.H.). Categories were used to group codes, which were then grouped into several themes. The categories and themes were shared with the other researchers in order to assess their validity.

## Results

**Fig. 1:**
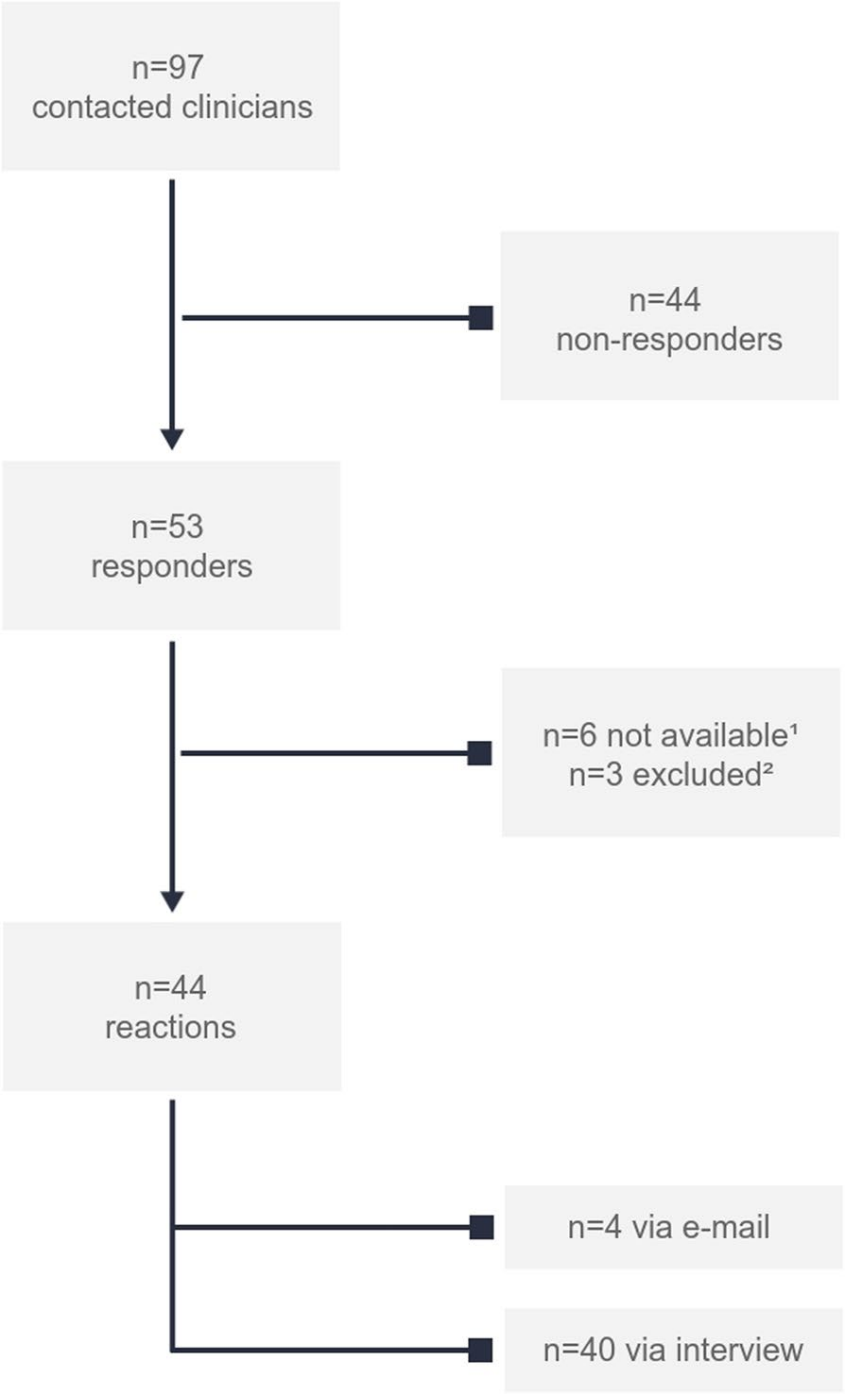
Selection of the clinicians. ^1^Six clinicians were not available due to lack of time*.* ^2^Three contacted clinicians were excluded since they no longer worked for the specific training region

A total of 97 eligible clinicians (radiologists; 10.3% and non-radiologists; 89.7%) were contacted by one of the researchers (F.H.) via e-mail between July and October 2020 (Table 2). Non-responders were excluded after a period of six months after the first e-mail was sent (*n* = 44). Clinicians were also excluded if they expressed to have no active involvement in medical curricula (*n* = 3) (Fig. 1). Of the included clinicians, four reactions were received via e-mail, while the other forty respondents provided their input by participating in an interview. The interviews lasted between 17 and 59 min. Participant characteristics are displayed in Table 2.

**Table 2:** The distribution of the contacted clinicians versus clinicians among the different medical specialties

**Medical specialty**	Contacted (n)		Participants (n)		
	*Total (n)*	*Distribution in study population (%)*	*Total (n)*	*Distribution in study population (%)*	*Response rate (%)*
Internal Medicine	**8**	*8.2%*	**4**	*9.1%*	*50%*
Neurology	**9**	*9,3%*	**3**	*6.8%*	*33.3%*
Surgery	**9**	*9.3%*	**6**	*13.6%*	*66.7%*
Pediatrics	**7**	*7.2%*	**3**	*6.8%*	*42.9%*
Emergency Medicine	**7**	*7.2%*	**4**	*9.1%*	*42.9%*
Obstetrics and Gynecology	**12**	*12.4%*	**4**	*9.1%*	*33.3%*
Ear-Nose-Throat Medicine	**8**	*8.2%*	**4**	*9.1%*	*50%*
Geriatrics	**9**	*9.3%*	**2**	*4.5%*	*22.2%*
General Practitioner	**18**	*18.6%*	**6**	*13.6%*	*33.3%*
Radiology and nuclear medicine	**10**	*10.3%*	**8**	*18.2%*	*80%*
***Total***	***97***		***44***		***45.4%***

The distribution of the contacted clinicians among the different medical specialties

**Fig. 2:**
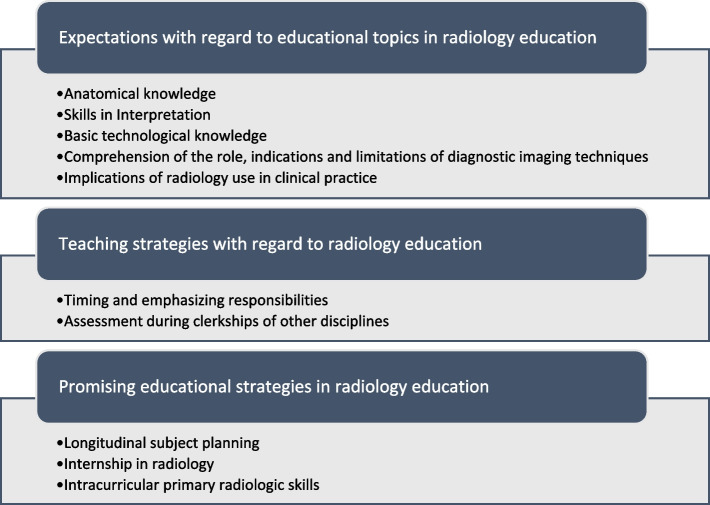
An overview of the subcategorized themes. Three themes accompanied by ten categories were derived from the interviews during the analysis after qualitative exploration of the opinions of clinicians and general practicioners on imaging technologies in medical school curricula

Ten categories of items were distilled from the transcribed codes, which were arranged in the following three themes (Fig. 2).

### Theme 1: Expectations with regard to educational topics in radiology education

#### Anatomical knowledge

Interviewees advocated that students need to be able to identify important anatomical landmarks and gross anatomical structures on the different radiologic imaging techniques. Knowledge of anatomy was believed to be the foundation of understanding a radiologic image by both radiologists and non-radiologists.

*“It starts with that [knowledge of the human anatomy], as this forms the foundation of radiology. Then, you can also start interpreting medical images” – General Practitioner*

*“… but I sense that there is little attention for forming an idea on the anatomical relations. And in the end, that is the essence…” – Surgeon*

Conversely, Computed Tomography scans (CT-scans) and Ultrasound (US) were suggested as ideal tools to teach anatomy in medical school. This was believed to benefit both anatomy education and radiology education. This combination provides clinical significance to anatomical structures as well as a three-dimensional insight into the anatomy. Furthermore, it would lead to early exposure to medical imaging in the curriculum. Magnetic Resonance Imaging scans (MRI-scans) were both suggested and dissuaded as a teaching tool because of their complexity.

#### Skills in interpretation

Interviews with both radiologists and non-radiologists revealed that the ability to interpret a wide range of radiological studies should not be included as a learning objective in medical school. Learning to interpret specific radiological studies (e.g., CT study of the thorax, brain MRI) should be incorporated in post-academic education for residents in training, as there is a greater exposure to these specific radiological studies during this period. Nevertheless, interviewees stated four things a medical student should be able to do concerning radiographs: (1) distinguish abnormal from normal (recognizing gross abnormalities), (2) identify some very common pathologies (e.g., pulmonary infiltrates, common bone fractures, joint luxation, pulmonary edema, hemorrhage, ischemia and malignancies), (3) identify acute diagnoses (e.g., vertebral fractures and pneumothorax on radiographs) and (4) acquire a systematic approach when reading radiographs (both chest radiographies and musculoskeletal radiographies). The extent to which these skills should be mastered under supervision was scarcely discussed and varied greatly.

#### Basic technological knowledge

Knowledge on the techniques of the four major different imaging modalities (radiography, CT, MRI and ultrasound) was regarded important as this provides knowledge on (contra-)indications and strengths and weaknesses of each imaging modality. It can also help a student interpret medical images as it helps to understand which structures are visible and why they are displayed in the way they are (e.g., the differences in size while comparing an AP- and PA-radiograph). It could also help students to understand the content of a radiological report (e.g., helping to understand why radiologists discuss patient positioning in their reports).

*“You have to know the basics. You can order radiographs, a CT-scan, or an ultrasound or an MRI-scan. And the reason why you would choose one option or another is always different, but you always visualize something with it. I consider it important to know what a specific imaging technique shows you.” – Emergency doctor.*

More specifically, it was considered important to have knowledge on the basics of ionization radiation, including its hazardous effects. For MR imaging, knowledge of the basic differences between T1-weighted-, T2-weighted-, and fluid-attenuation inversion recovery (FLAIR) sequences were disclosed as important subjects to master for medical students.

In addition, the impact that a radiological examination has on a patient (both mentally as well as physically) should also be embedded in the medical curriculum. This would also help future healthcare professionals to inform their patients properly in order to achieve well-informed consent.

*“...I think that it is good to know because we receive a lot of questions from patients about radiologic studies” – General Practitioner*

#### Comprehension of the role, indications and limitations of diagnostic imaging techniques

The most common (contra-)indications and limitations of the most frequently used modalities are seen as imperative knowledge which a student should acquire in medical school. This includes insights in accuracy rates of different radiological imaging methods and how these rates are influenced by other factors, as well as the costs of the different modalities. It should be noted that some clinicians mentioned that keeping up with the quickly changing indications could be a challenge and another participant did not find knowledge in indication important. All believed that you should always consult a radiologist when in doubt.

The benefits and drawbacks of the use of contrast agents, especially in CT imaging, and its (contra-)indications are worth emphasizing, for it has been mentioned multiple times in the interviews and is apart from one explicit modality.

*“I believe that it is very important that you know which radiological examinations are available and what you can use each one of them for. I also believe that it is very important that students are aware of the costs of the different imaging modalities and that they also take this into account when making a decision. And that they realize which study is useful for a specific question” – General Practitioner.*

#### Implications of radiology use in clinical practice

As each medical specialty has some level of experience with certain radiological imaging methods, it is important that students learn which techniques are used in various settings.. This was reported as a learning goal which should be achieved through experience-based learning (i.e., during internships). Also, clinicians expressed that it was paramount that students learn to write a concise though complete request for radiological imaging. In addition, students need to learn to look critically and should learn how to implement the radiologist's conclusion in the clinical setting for further medical management and/or follow-up.

Finally, students should also learn to consult the radiologist when questions arise regarding the most optimal imaging method or the radiological conclusion and how to interpret it.

*“Radiological findings are subjected to interpretation: someone sees an abnormality and expects it to be something. And those expectations are supported or undermined by the clinical presentation and you have to either provide this knowledge to the radiologist or have to take this into account yourself”—ENT-specialist.*

*“I noticed that they [students and junior doctors] have no comprehension of contrast agents and therefore just follow guidelines which state to ‘Check renal function’. They have no idea why and whether they have to order for contrast agents” – Radiologist.*

### Theme 2: Teaching strategies with regard to radiology education

#### Timing and emphasizing responsibilities

Most interviewees were convinced that during the Bachelor’s phase (i.e., the first three years of the university curriculum), imaging technology education needs to focus on the differences between modalities from a technical point of view. During those three years, radiological images should be used to help students understand the technical basis of imaging and recognize anatomical structures. This should gradually evolve into using radiological images to recognize simple pathology at the end of the Bachelor’s phase (e.g., bone fractures, pneumonia, pneumothorax). During the Master’s phase (last three years of university curriculum), the interviewees considered applied radiology as an important learning goal. This education could then be combined with recapitulating the anatomy.

*“I think that it should definitely be addressed in the Bachelor’s phase, but that the subjects in radiology that are embedded in an internship should be addressed in more detailed and specific way before that internship. I am actually getting thrilled by that idea”—General practitioner.*

It was believed that students will get more familiar with radiology when learning about imaging technologies is combined with anatomy and repeated over the years. Doing this while emphasizing different aspects of radiology during different learning phases of students, was also believed to result in a greater feeling of competence for medical students, especially with regard to chest radiographs and musculoskeletal radiographs. Therefore, radiology education during the Master’s phase of medicine should also focus on basic, structured interpretation of chest radiographs.

#### Assessment during internships of other disciplines

Interviewees suggested incorporating Entrusted Professional Abilities (EPAs) for radiology in the internships, so that radiology knowledge can be reviewed and improved continuously. Therefore, the knowledge of radiology can be monitored during the internships in the same way the discipline of radiology is integrated through all the different specialisms in medicine.

### Theme 3: Promising educational strategies in radiology education

#### Longitudinal subject planning

The idea of Longitudinal Learning Communities (LLCs) in radiology was discussed during all interviews. LLCs were defined as a community-based approach to learning during a time period of more than 1 year, encouraging meaningful student interaction and small-group learning as well as peer-group evaluation. LLCs were believed to help students to develop a collaborative approach to clinical practice, particularly in radiology. Clinicians believed that a timely repetition of anatomical and radiological knowledge before an internship would result in an improved learning experience.

Three clinicians, all non-radiologists, did not support more radiology education in already overcrowded medical curricula. One participant explicitly expressed that an LLC in radiology would take up too much time. Other interviewees (both radiologists and non-radiologists), however, considered radiology to be important enough to devote attention to, for example by use of LLCs. One participant also suggested saving time by combining the LLC with anatomy and physiology education throughout the medical curriculum. The learning materials used in such LLCs on radiology education were discussed as well. Suggested teaching methods included e-learnings and interactive workgroups. Additionally, the use of clinical cases during education as a form of applied radiology was expressed by many. Nevertheless, discrepancies remained with regard to the different teaching forms. Proposed forms were interactive teaching forms, clinical cases, lectures, computer orientated education, e-learnings, workshops, self-study, seminars, learning during the internships themselves (via specific educational moments, multidisciplinary meetings, during consulting hours at the outpatient department, radiology meetings, before surgery or via assignments). Clinicians expressed that they found it difficult to decide which educational methods would create the best learning environment for students.

Some additions to the described LLC were mentioned during the interviews. Several clinicians, both radiologists and non-radiologists, suggested adding practical ultrasound education to the LLC’s. One participant highlighted the importance of recapitulation shortly before practical education, also called in-time learning. This person believed that students would benefit more from good references, so they would know where to look when they need it and have clear learning objectives for radiology during their internships.

*‘…I strongly believe that just in time learning would be a valuable option. If you simply teach students in-time where to find specific knowledge on radiology, they will use it when they need it the most. Then, all they need to do is practice their knowledge” – Geriatrician.*

*‘If you learn about radiological examinations relevant in the clinical practice that you are about to embark in, you will learn the basics just prior to your internship and the clinical context will help you to complete the picture. Together, I would consider this a rich learning experience for students” – Radiologist.*

#### Internship in radiology

Due to a lack of time in the medical curriculum, most of the interviewees would not opt for the incorporation of a mandatory internship in radiology. Nevertheless, it was considered an important elective internship. Only one participant believed it was important to create time for a mandatory internship.

On the other hand, interviewees expressed that some practical experience in radiology for all medical students would be beneficial to: (1) gain insight into the tasks of a radiologist, (2) become aware of one’s own strengths and limitations regarding reading radiological examinations and (3) learn how to establish an optimal collaboration between radiologist and clinician. It was mentioned that such “intern days” could be integrated into the proposed LLCs in radiology or in various internships such as emergency and internal medicine or surgery.

*“I consider it a good idea to offer it as an internship for choice, apart from the LLC”- Internal medicine doctor*

#### Intracurricular primary radiologic skills

There was some discussion with regard to learning the skill of interpretation of a chest radiograph and the skill to perform a point-of-care ultrasound (POCUS). Chest radiography in itself takes a prominent place in radiology education and was believed to deserve a specific view on learning goals. There is an emerging use of POCUS in health care and the opinions on what should be taught on this subject diverse widely. Some interviewees thought that integrating POCUS as an intra-curricular learning goal would take up too much time to really let students master this skill. On the other hand, others were eager to implement teaching POCUS in the medical curriculum as it could serve as an extension on the physical examination with immediate results, low costs and high mobility with hand-held devices. It was mentioned that since so little is taught on ultrasound, there is so much to gain out of a bit more education.

*“If you ask me, we will all throw out our stethoscope and let everyone have an ultrasound machine and I do believe that time will come. I just do not know how soon” – Emergency doctor*

## Discussion

This study elucidated the views of both radiologists and non-radiologists and grouped these views in three themes: 1) Expectations with regard to educational topics in radiology education; 2) Teaching strategies with regard to radiology education; and 3) Promising educational strategies in radiology education.

These findings are largely corroborated by others. For example, Subramaniam et al. [53, 55, 55] also showed that radiology education should include the teaching of (contra-)indications for different imaging techniques, skills to systematically review chest and musculoskeletal radiographs, skills to identify gross abnormalities on radiographs and teaching students how to fit important findings in the clinical setting. However, contradictory to the studies of Subramaniam et al., interviewees did not express the reading of abdominal radiographs as an educational topic, which can be explained by the ongoing development of radiology in the clinical setting [2, 57]. At the time of the publication of the papers of Subramaniam et al., abdominal radiographs had a more prominent clinical role than today.

### Integration of radiology and anatomy education

Interviewees in this study stated that basic anatomical knowledge is needed to fully comprehend imaging studies. However, as less time is being assigned to anatomy education in medical curricula [18, 35, 4], learning about radiological examinations could become more complicated for students. Also, as Kourdioukova et al. [33] mention in their paper, Problem Based Curricula create a building block approach in which radiology and radiologic anatomy is relatively underrepresented in examinations. Integration of applied anatomy and applied radiology has been commonly suggested to optimize quality of anatomy and radiology education in modern medical curricula, [4, 14, 28, 36]. This was also objectified as radiology small group teaching significantly improved anatomy scores [8, 9] and radiology skills [40]. Additionally, combining radiology and anatomy education has been described to be easily implementable in existing preclinical curricula, because it requires few additional resources [62]. Integration of radiology education with other disciplines has also been suggested [42]. Interestingly, in the current study, MRI sequence which were considered basic knowledge comprised T1-weighted images, T2-weighted images and FLAIR images, whereas other sequences were not mentioned. Fat suppression techniques were not discussed, although several advantages are well-known in for example neuroradiology [56] and imaging of the musculoskeletal system [16]. Also, the use of diffusion-weighted imaging was not mentioned as part of the basic knowledge that a medical student must obtain. Possibly, clinicians omitted these sequence as the physical concepts are somewhat more complex to explain to students during rotations. However, the exact motives remain elusive. Together with the positive feedback to the LLC in the interviews of this study, a balanced integration of radiology education in various subjects of teaching could be a promising next step for radiology educators.

Other innovative teaching methods which might play a role in the future of anatomy and radiology education, such as augmented reality, virtual reality and combined use of these techniques with radiological data were not mentioned during the interviews. Nevertheless, several publications point out the possible advantages of each individual technique [6, 3, 12, 13, 41].

### Radiology education topics: reading chest radiographs and practical teaching of ultrasound skills

Although in this study chest radiographs were considered an important educational topic in medical school, there was a wide diversity in opinion to what extent a student should master this subcategory of imaging technologies. Even though this study was not able to provide results to what extent of supervision level or entrusted professional activity a medical student should master this skill, this study was the first that objectified this wide diversity in opinions. We believe this should be investigated more profoundly to be able to create a properly adjusted learning objective on this topic. Especially since Eisen and colleagues found that only 15% of their study population, consisting of medical students, interns, residents and fellows, felt capable to interpret chest radiographs independently in an academic medical center setting [19]. This lack of confidence has been found by others as well [7, 11].

Lastly, teaching ultrasound was a topic of debate in our study, which was widely discussed among the interviewees. This observed discrepancy is in line with literature on this topic [36, 50]. Although ultrasound has been described as an educational tool to improve anatomy knowledge, physical examination skills, increase diagnostic accuracy and advance patient safety, the evidence regarding the effects of ultrasound education on these outcomes is very limited [20]. Nevertheless, various studies reported that medical students consider ultrasound education as valuable [15, 17, 46, 49, 59]. Despite this increased demand of ultrasound education in medical school, studies showed that hands-on education of ultrasound is taught at a minority of universities in Europe and the United States [43, 45]. More research is needed to either create insight into the learning objectives of ultrasound in medical curricula or to chart the potential benefits of teaching ultrasound in medical school. Additionally, the effects of using ultrasound for educational goals on learning outcomes should be studied as well.

### Strengths and limitations

The major strength of this study was the qualitative study design as a recent review highlighted that quality research is needed to investigate when and how radiology should be included in medical education [8, 9]. A second strength concerns the exploration of the thoughts and opinions of a wide variety of clinicians included in this study. The sparse availability of recent scientific literature on the teaching of a dynamic subject like medical imaging illustrates that this is a relatively understudied domain and, simultaneously, shows the importance of the present work. This work, however, is not without its limitations. One limitation of this study was formed by the strictly defined inclusion criteria which only allowed clinicians from one region within The Netherlands to participate. The ideas on this topic within this region can differ from others since every training region has its own personal and cultural view on certain subjects and specific spearheads. This limitation regarding generalizability of the reported themes might also exist for the clinicians’ views on radiology education in countries other than The Netherlands. Secondly, this study population cannot be considered as a generalizable population of clinicians which are involved in medical education. For example, the number of radiologists participating in this study was larger as compared to the number of general practitioners (Table 2). Therefore, radiologists were overrepresented in the study population. In addition, some medical disciplines, such as psychiatry, ophthalmology and dermatology were excluded from this interview study due to the fact that these clinicians do not frequently encounter radiology. However, the risk of potential bias is limited as the nature of this study and research question did not warrant the inclusion of these clinicians. Furthermore, a limitation of the qualitative study design concerns the relative subjectivity of the results as participants hold control over the content of the data. This prevents quantification of the results and warrants future studies to investigate the statistical significance of the here described findings [10, 38]. Additionally, it must be noted that clinicians are no education experts. Future implementation of these results should be carried out in close collaboration with education experts.

## Conclusions

This qualitative study provided more in-depth knowledge on well-known topics with regard to radiology education in medical curricula. More knowledge with regard to educational methods, timing of radiology education was distilled and several new topics arose. This includes thoughts on educating ultrasound skills to undergraduates and the views on a longitudinal learning community in radiology in order to integrate imaging technologies in a problem based medical curriculum. It was recommended that radiology education should be more embedded in the medical curriculum and various educational strategies and topics to achieve this were suggested. Nevertheless, to which extent these educational topics should be mastered, what resulting learning objectives will need to entail and how to evaluate them need further research.

## Data Availability

The dataset generated from the interviews and analyzed during the current study are not publicly available since individual privacy could potentially be compromised but are available from the corresponding author on reasonable request.

## Abbreviation

LLC: Longitudinal learning community/curriculum
